# microRNAs and genetic diseases

**DOI:** 10.1186/1755-8417-2-7

**Published:** 2009-11-04

**Authors:** Nicola Meola, Vincenzo Alessandro Gennarino, Sandro Banfi

**Affiliations:** 1 Telethon Institute of Genetics and Medicine (TIGEM), 80131 Naples, Italy

## Abstract

microRNAs (miRNAs) are a class of small RNAs (19-25 nucleotides in length) processed from double-stranded hairpin precursors. They negatively regulate gene expression in animals, by binding, with imperfect base pairing, to target sites in messenger RNAs (usually in 3' untranslated regions) thereby either reducing translational efficiency or determining transcript degradation. Considering that each miRNA can regulate, on average, the expression of approximately several hundred target genes, the miRNA apparatus can participate in the control of the gene expression of a large quota of mammalian transcriptomes and proteomes. As a consequence, miRNAs are expected to regulate various developmental and physiological processes, such as the development and function of many tissue and organs. Due to the strong impact of miRNAs on the biological processes, it is expected that mutations affecting miRNA function have a pathogenic role in human genetic diseases, similar to protein-coding genes. In this review, we provide an overview of the evidence available to date which support the pathogenic role of miRNAs in human genetic diseases. We will first describe the main types of mutation mechanisms affecting miRNA function that can result in human genetic disorders, namely: (1) mutations affecting miRNA sequences; (2) mutations in the recognition sites for miRNAs harboured in target mRNAs; and (3) mutations in genes that participate in the general processes of miRNA processing and function. Finally, we will also describe the results of recent studies, mostly based on animal models, indicating the phenotypic consequences of miRNA alterations on the function of several tissues and organs. These studies suggest that the spectrum of genetic diseases possibly caused by mutations in miRNAs is wide and is only starting to be unravelled.

## The microRNA world

microRNAs (miRNAs) are a class of single-stranded RNAs (ssRNAs), 19-25 nucleotides (nt) in length, generated from hairpin-shaped transcripts. They control the expression levels of their target genes through an imperfect pairing with target messenger RNAs (mRNAs), mostly in their 3' untranslated regions (3' UTRs) [1]. The biogenesis of miRNAs involves a complex protein system that includes members of the Argonaute family, Pol II-dependent transcription and the two RNase III proteins, Drosha and Dicer [2]. miRNAs are first transcribed in the nucleus as long transcripts, known as primary miRNA transcripts (pri-miRNAs), which can sometimes contain multiple miRNAs [3,4]. Few pri-miRNA transcripts have been studied in detail, but increasing evidence suggests that miRNAs are regulated and transcribed like protein encoding genes [5].

In brief, within the nucleus, Drosha first forms a micro-processor complex with the double-stranded RNA-binding protein DGCR8 [6]. It then processes the pri-miRNAs into a smaller, stem-loop miRNA precursor of ~70 nucleotides (pre-miRNA) [7]. pre-miRNAs are exported, in turn, across the nuclear membrane and into the cytoplasm by the Exportin-5 complex [8-10]. These pre-miRNAs are further cleaved by Dicer thus producing a 19- to 25-nucleotide RNA duplex. These duplexes are then incorporated into a ribonucleoprotein complex (RNP) called RISC-like complex [11,12], referred to as the miRNA-induced silencing complex (miRISC). Only one strand of the miRNA-duplex, known as the mature miRNA, is incorporated into the miRISC complex, while the other strand, the miRNA-star (miRNA*), is degraded [1] although, recently, miRNAs* have been found to play a role similar to that of their cognate miRNAs. Within the miRISC complex, miRNAs bind to the mRNA targets and regulate gene expression, either at the translational level [13,14] or at the transcript level [15-17] or both [18]. A crucial role in the recognition of the target mRNA by the miRNA is played by the so-called seed region, which is composed of six to seven nt, which shows a perfect complementarity between a miRNA and its target. miRNA can be localized in the intergenic (40%) or the intragenic (60%) regions [19]. Intragenic miRNAs are located within other transcriptional units which are termed host genes. The vast majority of intragenic miRNAs is localized within the intronic regions of their host genes and only a minority (10%) lies within exonic regions, usually pertaining to the non protein-coding host genes. Interestingly, it has been demonstrated that many intronic miRNAs and their host genes are co-regulated and co-transcribed from a common promoter [20-22].

## miRNAs and their implication in human diseases

miRNAs are implicated in a wide range of basic biological processes, including development, differentiation, apoptosis and proliferation [23,24]. Since the discovery of the strong impact of miRNAs on biological processes, it has been hypothesized that mutations affecting miRNA function may have a pathogenic role in human diseases. A large body of evidence has already shown that aberrant miRNA expression is implicated in most forms of human cancer [25-27], but fewer studies have established a clear link between miRNAs and human genetic disorders. Initially, there were two main (and contrasting) arguments against the hypothesis of miRNAs as genes responsible for human genetic diseases: (1) each miRNA is endowed with such a basic role in the regulation of gene expression and consequently in the regulation of basic cellular processes that a significant alteration of their function is not compatible with cell survival and ultimately with life; and (2) considering the great deal of redundancy in miRNA actions, a significant alteration of the function of a single miRNA may only give rise to subtle modifications in both the cellular transcriptome and proteome, which are unable to determine a significant perturbation of biological processes and ultimately lead to a diseased phenotype.

Our aim in this review is to provide an overview of the evidence available to date which support the pathogenic role of miRNAs in human genetic diseases, with a particular focus on monogenic disorders. In order to achieve this goal, we will first describe the types of mutations affecting miRNA function that can result in human monogenic disorders, giving some recently described examples. In the second part of this review, we will give a broader picture of the hypothetic involvement of miRNAs in the pathogenesis of human monogenic diseases based on the results obtained *in vivo *from the analysis of several animal models characterized by either the global perturbation of miRNA pathways or by the perturbation (either inactivation or overexpression) of single miRNAs.

## Types of miRNA mutations with a pathogenic role in human mendelian disorders

**Figure 1:**
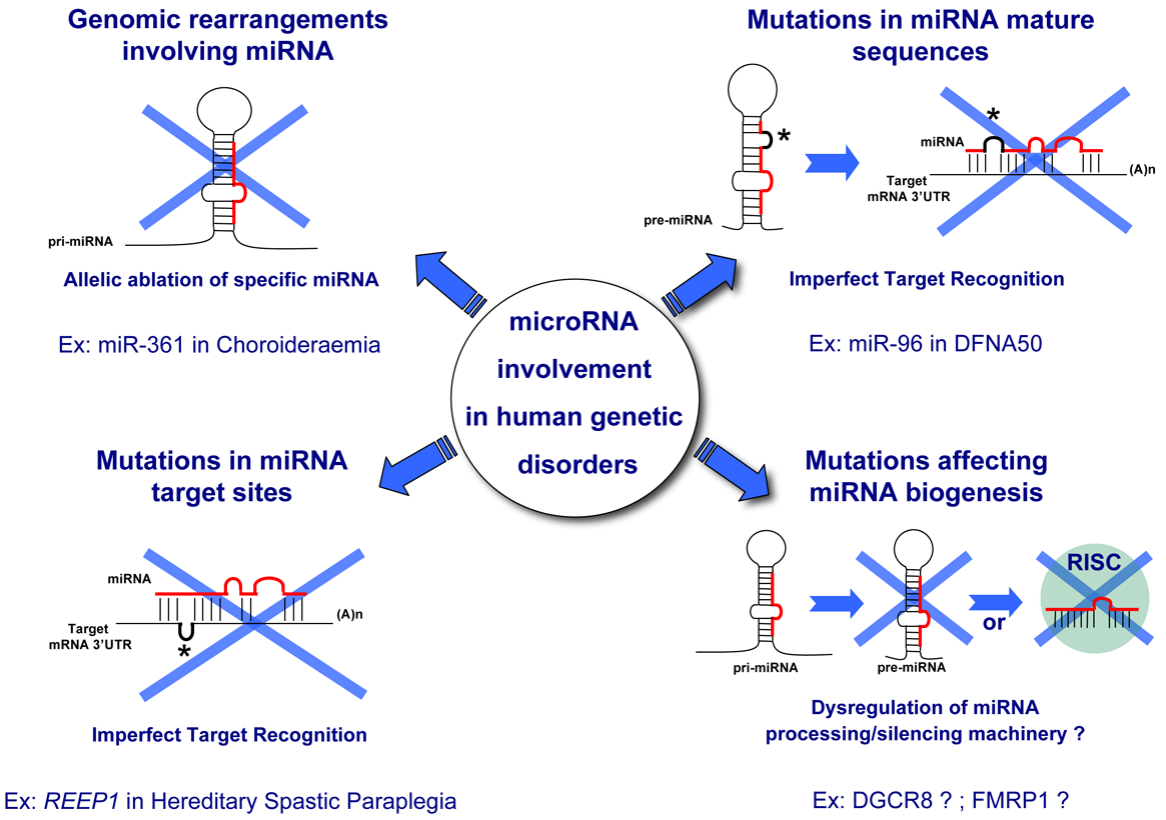
**Schematic diagram summarizing the main types of miRNA mutations with a potential aetiopathogenic role in monogenic disorders (see text for further details)**.

Given the mechanisms of action of miRNAs (see above), three main types of mutation mechanisms affecting miRNA function can be envisaged (Figure 1): (1) mutations affecting primarily miRNAs, either point mutations in the mature sequence or larger mutations (that is, deletions or duplications of the entire miRNA locus); (2) mutations in the 3' UTR of mRNAs that can lead to the removal or to the *de novo *generation of a target recognition site for a specific miRNA; and (3) mutations in genes which participate in the general processes of miRNA processing and function and, therefore, are predicted to impact on global miRNA function.

## Mutations affecting primarily miRNAs

### Large mutations

Likewise, protein-coding loci and also miRNA loci can be subjected to large mutations, such as deletions or duplications. To date, there are no examples of such mutations which are clearly associated with human mendelian diseases. However, a careful analysis of the genomic organization of miRNAs reveals that a number of intragenic miRNAs are localized within host genes (see above) whose mutations are responsible for human genetic disorders. By analysing the mutation spectrum previously described for the latter disease genes in the Human Gene Mutation Database (HGMD) [28], we found evidence that some mutations do, indeed, significantly affect one or more miRNAs (Table 1). This is the case, for instance, in certain intragenic deletions responsible for Duchenne muscular dystrophy, choroideraemia and Dent disease, among others, which are also predicted to encompass some miRNAs. It is of crucial importance to confirm these predictions and to determine whether or not the deletion of these miRNAs is able to play a role in the phenotype observed. Furthermore, several miRNAs loci are also either deleted or duplicated (Table 1) in some well-known human aneuploidy syndromes, and there is initial evidence of their contribution to the pathogenic mechanisms of the complex manifestations of these disorders [29].

**Table 1:** Example of LARGE mutations, responsible for human genetic diseases, encompassing microRNA (miRNA) loci

Disease	OMIM	Inheritance pattern	Responsible gene/locus	Disease gene locus	miRNA involved in the mutation	Type of mutation involving the miRNA	Selected references
Choroideraemia	*300390	X-linked recessive	CHM	Xq21.2	miR-361	Complete miRNA deletion	[106,107]
							
Dent disease	#300009	X-linked recessive	CLCN5	Xp11.22	miR-500; miR-600; miR-188; miR-362-5p; miR-362-3p; miR-500; miR-501; miR-502; miR-532-5p; miR-532-3p	Complete miRNA deletion	[108]
							
Epidermolysis bullosa	#226650	Autosomal recessive	COL17A1	10q24.3	miR-936	Complete miRNA deletion (one allele)	[109,110]
							
Muscular dystrophy, Duchenne/Beker	#310200; #300376	X-linked recessive	DMD	Xp21.1	miR-548f-5	Complete miRNA deletion	[111-113]
							
Haemophilia A	+306700	X-linked recessive	F8	Xq28	miR-1184	Complete miRNA deletion	[114-116]
							
Beckwith-Wiedemann syndrome	#130650	Autosomal dominant	H19	11p15.5	miR-675	Complete miRNA deletion	[117]
							
Pantothenate kinase-associated neurodegeneration/Harp syndrome	#234200; #607236	Autosomal recessive	PANK2	20p13-p12.3	miR-103-2	Complete miRNA deletion	[118]
							
Polycystic kidney disease 1	#173900	Autosomal dominant	PKD1	16p13.3-p13.12	miR-1225	Complete miRNA deletion	[119]
							
HHH syndrome	#238970	Autosomal recessive	SLC25A15	13q14	miR-621	Complete miRNA deletion (one allele)	[120]
							
Down syndrome	#190685	---	Chromosome 21 trisomy	Chromosome 21	miR-99a; let-7c; miR-125b; miR-155; miR-802	miRNA triplication	[121-123]
							
Williams-Beuren syndrome	#194050	Autosomal dominant	7q11.23 deletion	7q11.23	miR-590	Complete miRNA deletion	[124,125]
							
DiGeorge syndrome	#188400	Autosomal dominant	22q11.2 deletion	22q11.2	miR-648; miR-185; miR-1306; miR-1286; miR-649; miR-301b; miR-130b; miR-650	Complete miRNA deletion	[126-128]

### Point mutations in miRNA mature sequences

Duan and colleagues, in 2007, described a single nucleotide polymorphism (SNP) within the seed region of miR-125a. Through a series of *in vitro *analyses, the authors demonstrated that this SNP in miR-125a, in addition to reducing miRNA-mediated translational suppression, significantly altered the processing from pri-miRNA to pre-miRNA. Although this SNP has not been associated with a disease status, these data suggest, for the first time, that SNPs that reside within miRNA genes may, indeed, impair miRNA biogenesis and alter target selection and, therefore, have a potentially profound biological effect [30].

The first example of point mutations in the mature sequence of a miRNA with an aetiopathogenic role in a human mendelian disease has been recently reported by Mencìa *et al*. [31]. They identified two different nucleotide substitutions in the seed region of the human miR-96 in two Spanish families affected by an autosomal dominant form of deafness, namely DFNA50. In particular, both the mutations, miR-96 (+13G>A) and (+14C>A), which were not present in several unrelated normal-hearing Spanish controls, were segregated in both of the families with a hearing impairment. miR-96, together with miR-182 and miR-183, is transcribed as a single polycistronic transcript and is reported to be expressed in the inner ear. For this reason, the authors also carried out a mutation screening of miR-182 and miR-183 in the same cohort of patients, tested for miR-96. However, they did not find any potential mutation, although this does not exclude the possibility that the latter two miRNAs may be involved in the pathogenesis of other forms of deafness. The fact that both the above families manifested the hearing loss postlingually indicated that probably neither of the two miR-96 mutations resulted in impaired development of the inner ear. Instead, they could have had an impact on the regulatory role that miR-96 plays in the hair cells of the adult cochlea which maintain the gene expression profiles required for its normal function. *In vitro *experiments showed that both mutations impaired, but did not abrogate, the processing of miR-96 to its mature form, although an additional indirect effect on the expression of miR-182 and miR-183 due to the miR-96 mutations cannot be excluded. Furthermore, a luciferase reporter assay confirmed that both mutations were able to affect the targeting of a subset of selected miR-96 target genes, mostly expressed in the inner ear. In contrast, no significant gain of function was associated with these two mutations, at least for the potentially new acquired miR-96 targets investigated. In addition, after an ophthalmologic revision, no ocular phenotype was observed in individuals carrying mutations in miR-96 (age range between 2 and 66 years), suggesting that its specific targets in the retina, a site in which miR-96 is also strongly expressed, were not critical for its function or that the translation of these targets was not markedly affected [31].

The finding of a single base change (A>T) in the seed region of miR-96 in a mouse mutant (*diminuendo*) with a progressive hearing loss phenotype, provided additional support to the finding that a single base change in miR-96 is the causative mutation behind the hearing loss phenotype in both man and mouse [32]. In particular, the *diminuendo *mutant showed progressive hearing impairment in heterozygotes and profound deafness in homozygotes associated with hair cell defects. Lewis and colleagues suggested that the degeneration observed in homozygotes could be a consequence of a prior dysfunction of the hair cells. Bioinformatic analysis indicated that the mutation has a direct effect on the expression of many genes, including transcription factor genes, that are directly required for hair cell development and survival. The large number of genes whose expression is affected by miR-96 suggests that the mechanism that explains the effects of the mutation may not be simple but, rather, may be the result of a combination of different small effects that act in concert to cause hair cell dysfunction [32].

### Mutations in miRNA target sites

In animal cells, most miRNAs form imperfect hybrids with sequences in the 3'-UTR, with the miRNA 5'-proximal 'seed' region (positions 2-8) providing most of the pairing specificity [33,34]. However, evidence is also accumulating that miRNAs may target mRNA-coding regions [35]. Generally, miRNAs inhibit protein synthesis either by repressing translation or by bringing about deadenylation and degradation of mRNA targets [36]. Since more than 700 miRNAs have been identified in the human and mouse genomes [37], and also considering that each miRNA can regulate, on average, the expression of 100-200 target genes [38,39], the whole miRNA apparatus seems to participate in the control of the gene expression for a significant proportion of the mammalian gene complement.

It is conceivable that some sequence variations falling within the 3'-UTR of mRNA may alter miRNA recognition sites, either by altering functional miRNA target sites or by creating aberrant miRNA target sites. Both types of sequence variations may potentially have deleterious effects in the case of either miRNA-mRNA pairs endowed with a biologically relevant (and non-redundant) role or when the formation of an illegitimate miRNA target occurs in mRNAs that are under selective pressure to avoid target sites for that particular miRNA (that is, in the case of the so-called anti-targets) [40].

One of the first animal disorders with a mendelian transmission reported to be caused by dysregulation of a specific miRNA-mRNA target pair was the Texel sheep model. The Texel sheep phenotype is characterized by an inherited muscular hypertrophy that is more pronounced in the hindquarters of sheep [41]. Clop *et al*. [41] demonstrated that the *myostatin *(*GDF8*) gene of Texel sheep is characterized by a G to A transition in the 3' UTR that creates a target site for mir-1 and mir-206, which are highly expressed in the skeletal muscle. This sequence change leads to a translational inhibition of the *myostatin *gene and, hence, is responsible for the muscular hypertrophy of Texel sheep [41].

There are now some examples of sequence variations in the 3'-UTR of mRNAs altering miRNA recognition sites which have been suggested to have a pathogenic role in human genetic diseases. The first was reported by Abelson *et al*. [42], who identified two independent occurrences of the identical sequence variant in the binding site for the miRNA hsa-miR-189 (now termed miR-24*) in the 3'-UTR of the *SLITRK1 *mRNA in familial cases of Tourette's syndrome, a developmental neuropsychiatric disorder characterized by chronic vocal and motor tics. This 3'-UTR sequence variation in *SLITRK1 *was proposed in order to determine an increased extent of repression of this gene by hsa-miR-189 (miR-24*). It must be underlined, however, that the involvement of *SLITRK1 *in Tourette's syndrome has been subsequently questioned by other reports [43-47]. The second example is represented by two different point mutations in the 3'-UTR of the *REEP1 *gene which have been associated with an autosomal dominant form of hereditary spastic paraplegia (SPG31) [48,49]. These mutations, which alter the sequence of a predicted target site for miR-140, were found to segregate with the disease phenotype and were not detected in a large set of human controls. These data strongly suggest the pathogenic role of the impaired miR-140-*REEP1 *binding in some SPG31 families, although so far no functional data have been provided to consolidate this hypothesis.

Georges and colleagues tried to address, in a more systematic way, the potential implications of sequence variations in the 3'-UTR of mRNAs in the pathogenesis of human diseases. They demonstrated, through SNP analysis, that sequence variations creating or destroying putative miRNA target sites are abundant in the human genome and suggested that they might be important effectors of phenotypic variation [50]. A list of additional sequence variations altering putative miRNA recognition sites, and with a potential role in human disease, can be found in a review by Sethupathy and Collins [51]. The authors critically reviewed a number of studies that claimed that there is an association between the presence of polymorphisms/mutations in miRNA target sites (poly-miRTSs) and human diseases, giving a special emphasis on possible biases and confounding factors. They concluded that only a few presented rigorous genetic and functional evidence. The authors therefore suggested a set of concrete recommendations in order to guide future investigations of putative disease-associated poly-miRTSs [51].

### Mutations impacting on global miRNA function

As previously described, a number of different proteins are involved in the processing of miRNAs. Mutations altering the function of these proteins are predicted to determine a global alteration of miRNA function. This aspect is exploited, for instance, in the experimental inactivation of *Dicer *that is used to assess the biological consequences of the global perturbation of miRNA activity in whole organisms or specific tissues/cell types (see also below). Complete loss-of-function mutations of certain key members of the miRNA processing pathway (such as Drosha and Dicer) are expected to be incompatible with life and, therefore, are not believed to play a role in the pathogenesis of human monogenic disorders. However, there are two human diseases characterized by mutations in genes involved in miRNA processing/activity, namely DiGeorge syndrome and Fragile X syndrome. The *DGCR8 *gene, which maps to chromosomal region 22q11.2, is commonly deleted in DiGeorge syndrome [52], characterized by cardiovascular defects, craniofacial defects, immunodeficiency and neurobehavioral alterations. As previously mentioned, DGCR8 is a component of the Drosha complex and its haploinsufficiency in DiGeorge syndrome patients may have a potential impact on miRNA processing. However, also based on the results of the targeted inactivation of the corresponding gene in mouse [53], there are no data, thus far, which point to a functional effect of DGCR8 haploinsufficiency on miRNA biogenesis.

The second example is represented by the Fragile X syndrome. The product of the *FRM1 *gene, whose loss-of-function is responsible for this condition, is a selective RNA binding protein. It has been proposed that the FMRP1 protein may function as a translational repressor of its mRNA targets at synapses by recruiting the RISC complex along with miRNAs and by facilitating the recognition between miRNAs and a specific subset of their mRNA targets. This interaction is suggested to be important in the process of synaptic plasticity which, instead, is largely compromised in Fragile X syndrome patients [54,55]. However, this hypothesis requires further investigations. In conclusion, there is no evidence so far to support a direct role of altered global miRNA processing in human hereditary disorders.

## What other human genetic diseases are potentially caused by miRNA dysfunction?

The number of cases in which mutations in miRNA and in miRNA targets have proven to be firmly associated with monogenic disorders is still limited (see above). However, we expect the contribution of miRNAs, and related pathways to the pathogenesis of these conditions, to increase in the near future, following both a better knowledge of their biological function and the advancement of high-throughput mutation detection approaches [56-58]. We will now try to make some hypotheses on the possible diseases in which miRNAs may have a pathogenic role, mainly based on the results obtained from the analysis of animal models.

## microRNAs and heart 'diseases'

The heart is the first organ to form and to function during vertebrate embryogenesis [59]. Perturbations in normal cardiac development and function result in a variety of cardiovascular diseases, which are the leading cause of death in developed countries [60].

The first indication of the global involvement of miRNAs in heart development and function was derived from the analysis of conditional knockout mice carrying a cardiac-specific inactivation of the Dicer enzyme. As described above, Dicer plays a key role in miRNA biogenesis and its inactivation is predicted to cause a general deficiency of the mature forms of all miRNAs.

Chen and colleagues reported that cardiac-specific knockout of the *Dicer *gene led to rapidly progressive dilated cardiomyopathy, heart failure and postnatal lethality. *Dicer *mutant mice showed misexpression of cardiac contractile proteins and profound sarcomere disorder. Functional analyses indicated significantly reduced heart rates and a decreased fractional shortening in *Dicer *mutant hearts. Furthermore, this study demonstrated, for the first time, the essential role of Dicer in cardiac contraction and also indicated that miRNAs play a critical role both in normal cardiac function and under pathological conditions [61].

Moreover, da Costa and colleagues found that an inducible deletion of *Dicer *in the adult mouse heart results in a severe alteration of myocardial histopathology, suggesting a crucial role for this enzyme in ensuring the integrity of the postnatal heart. Interestingly, *Dicer *depletion in the juvenile heart provoked an overall tendency to arrhythmogenesis and less marked myocyte hypertrophy, but its inactivation in the adult myocardium gave rise to myocyte hypertrophy and angiogenic defects. These findings seem to imply the presence of differences in the biological role, as a whole, of miRNAs between the juvenile and the adult myocardium [62].

The generation of cardiac disease-like phenotypes in animal models may not only be caused by a global alteration of miRNA function but also by the dysfunction of specific miRNAs. For instance, miR-1-2 appears to be involved in the regulation of diverse cardiac and skeletal muscle functions, including cellular proliferation, differentiation, cardiomyocyte hypertrophy, cardiac conduction and arrhythmias [63]. miR-1, together with another heart-specific miRNA (miR-133a), is known to be transcribed by a duplicated bicistronic genetic locus (miR-1-1/miR133a-2 and miR-1-2/miR133a-1) sharing identical sequences of the mature miRNAs. Mice lacking miR-1-2 present a spectrum of abnormalities, ranging from ventricular septal defects and early lethality to cardiac rhythm disturbances. These mice also featured a striking cell-cycle abnormality in myocytes, leading to hyperplasia of the heart with nuclear division persisting postnatally. Remarkably, the persistence in these mice of the other identical copy of miR-1-2 (that is, miR-1-1) did not compensate for the loss of miR-1-2, at least for many aspects of its function. While it is likely that mice lacking both miR-1-1 and miR-1-2 will have increasingly severe abnormalities, the range of defects upon the deletion of miR-1-2 highlighted the ability of miRNAs to regulate multiple diverse targets *in vivo *[63]. The subtle dysregulation of numerous developmental genes may contribute to the embryonic defects observed in miR-1-2 mutants. These included: (1) *Hrt2*/*Hey2*, a member of the Hairy family of transcriptional repressors that mediates Notch signalling, which can itself cause heart disease [64]; and (2) *Hand1*, a bHLH transcription factor involved in ventricular development and septation that, in combination with *Hand2 *(a paralog of *Hand1*), is known to regulate expansion of the embryonic cardiac ventricles in a gene dosage-dependent manner [65]. Furthermore, in miR-1-2 mutants, the observed abnormality in the propagation of cardiac electrical activity, despite normal anatomy and function, was correlated with the upregulation of the direct target *Irx5*, a transcription factor, resulting in ventricular repolarization abnormalities and a predisposition to arrhythmias.

Jiang *et al*. added one more piece to the puzzle represented by the miR-1/miR-133a cluster. They extensively characterized genetically engineered mice deficient for either miR-133a-1, or miR-133a-2, or both as well as mice overexpressing miR-133a [66]. While miR-133a-1 and miR-133a-2 seemed to have redundant functions and did not cause obvious cardiac abnormalities when deleted individually, targeted deletion of both miRNAs resulted in cardiac malformations and embryonic or postnatal lethality. miR-133a double knockout mice displayed two distinct lethal phenotypes: (1) either large ventricular sept defects (VSDs), dilated right ventricles, and atria leading to death shortly after birth; or (2) survival into adulthood, no VSDs but dilated cardiomyopathy (DCM), cardiac fibrosis and heart failure. Surprisingly, miR-133a deficiency did not lead to hypertrophic cardiomyopathy, as one would have been expected from previous studies, in which miR-133a-antagomir treatment induced cardiac hypertrophy in mice [67]. Several genes involved in cardiomyocyte cell cycle control, such as *Cyclin D1*, *Cyclin D2 *and *Cyclin B1*, were found to be significantly upregulated in miR-133a deficient hearts as well as several smooth muscle genes, such as *smooth muscle-Actin*, *Transgelins*, *Calponin I *and the myogenic transcription factor *SRF*.

In a further attempt to dissect the effects of miR-133a on cardiomyocyte proliferation, Liu *et al*. [68] overexpressed miR-133a under the control of the cardiac β-*myosin heavy chain *promoter. Surprisingly, transgenic animals died by embryonic day 15.5 (E15.5) due to VSDs and diminished cardiomyocyte proliferation, which resulted in ventricular walls only consisting of two to three cell layers that were unable to fulfill the hemodynamic needs of the developing mouse [66].

Morton *et al*. reported that miR-138 is expressed in specific domains of the zebrafish heart and is required to establish appropriate chamber-specific gene expression patterns. Disruption of miR-138 function led to expansion in the expression of ventricular-specific genes, normally restricted to the atrio-ventricular valve region, and, ultimately, to disrupted ventricular cardiomyocyte morphology and cardiac function. Furthermore, the authors demonstrated that miR-138 helps in establishing discrete domains of gene expression during cardiac morphogenesis by targeting multiple members of the retinoic acid signaling pathway [69].

van Rooij and colleagues described cardiac hypertrophy and failure in a mouse model overexpressing mir-195, which is generally upregulated in hypertrophic human hearts [70]. Overexpression of miR-195 under the control of the α-*myosin heavy chain *(*Mhc*) promoter initially induced cardiac growth with disorganization of cardiomyocytes, which progressed to a dilated heart phenotype by 6 weeks of age. More striking was the dramatic increase in size of individual cardiomyocytes in miR-195 transgenic mice compared to normal mice. Furthermore, ratios of heart weight to body weight were also dramatically increased in miR-195 transgenic (Tg) animals as compared to wild-type littermates, indicating that overexpression of miR-195 was sufficient to stimulate cardiac growth. Thus, the cardiac remodelling induced in the miR-195 Tg animals was specifically caused by the functional effects of this miRNA rather than a general nonspecific effect resulting from miRNA overexpression, suggesting that increased expression of miR-195 induced hypertrophic signalling, leading to cardiac failure.

Based on all the previously described findings it is tempting to speculate a possible role of specific miRNAs in human genetic forms of heart hypertrophy and failure. This hypothesis is also supported by the dysregulation of miRNA expression observed in several cardiovascular diseases in man [71-73].

## microRNAs and central nervous system (CNS) 'diseases'

Multiple lines of evidence indicate the potential role of miRNAs in neuronal cell development and maturation. Both the mouse and human brain express a large spectrum of distinct miRNAs compared with other organs [74,75]. Therefore, the implications of dysregulation of miRNA networks in human diseases affecting the CNS are potentially enormous.

In recent years, different conditional *Dicer *null mouse lines in the brain have been generated. They have provided initial insight into the *in vivo *role of miRNAs in the mammalian CNS and particularly in the neuronal maintenance of the mouse brain [76-79].

Schaefer and colleagues [76] described the phenotypic characterization of *Dicer *null mice in Purkinje cells of the cerebellum. They performed the inactivation of *Dicer *exclusively in the post-mitotic Purkinje cells by using the Purkinje cell-specific *Pcp2 *promoter-driven Cre recombinase. This inactivation led to the relatively rapid disappearance of cerebellar-expressed miRNAs followed by a slow degeneration of Purkinje cells. The loss of *Dicer *and the decay of miRNAs had no immediate impact on Purkinje cell function, as assessed by electrophysiological studies and analysis of animal locomotion. However, the continuous lack of miRNAs led eventually to Purkinje cell death and ataxia, suggesting that a long-term absence of miRNAs in these cells results in a neurodegenerative process [76].

In a recent work, the inactivation of *Dicer *in the cortex and hippocampus beginning at embryonic day 15.5 resulted in dramatic effects on cellular and tissue morphology and led to gross brain malformations including microcephaly, increased brain ventricle size, and reduction in size of white matter tracts, leading to an early postnatal death [80]. Furthermore, mutant mice were ataxic with visible tremors during motility. Ataxic gait was detected by postnatal day 14-15, but often occurred as early as postnatal day 12. *Dicer *mutant animals also displayed hind limb crossing, typical of animals with motor impairment. Therefore, loss of miRNA function in some mouse brain regions during late development results in a significantly decreased lifespan as a consequence of severe malformations as well as motor impairments due to an increased cortical apoptosis [77].

To determine the role of miRNAs in dopaminoceptive neurons, Cuellar *et al*. ablated *Dicer *in mice by using a dopamine receptor-1 (*Dr-1*) promoter-driven Cre. The mutant animals displayed a range of phenotypes including ataxia, front and hind limb clasping, reduced brain size and smaller neurons. Surprisingly, dopaminoceptive neurons without Dicer survived during the life of the animal in contrast with other mouse models in which neurodegeneration was observed in the absence of Dicer [77].

miRNAs have also been studied in early neurogenesis during the development of the mammalian cerebral cortex and the switch of neural stem and progenitor cells from proliferation to differentiation. *Dicer *ablation in neuroepithelial cells at embryonic day (E) 9.5 resulted in massive hypotrophy of the postnatal cortex and death of the mice shortly after weaning. Remarkably, the primary target cells of *Dicer *ablation, the neuroepithelial cells and the neurogenic progenitors derived from them, were unaffected by miRNA depletion with regard to cell cycle progression, cell division, differentiation and viability during the early stage of neurogenesis and only underwent apoptosis starting at E14.5, suggesting that progenitors are less dependent on miRNAs than their differentiated progeny [79].

miRNA function was studied also for another part of the CNS (that is, the retina). Damiani *et al*. described a partial ablation of *Dicer *in the developing mouse retina by using a Cre line under the *Chx10 *promoter, a gene mostly expressed in retinal progenitors and specific adult retinal interneuronal cells. These mice apparently showed no visible impact on early postnatal retinal structure and function. Retinal lamination appeared normal and all expected retinal cell types were represented. However, as observed for the other *Dicer *null mutants, progressive and widespread structural and functional abnormalities were detected, culminating in loss of photoreceptor-mediated responses to light and extensive retinal degeneration [78]. Therefore, the observation that progressive retinal degeneration occurred after removal of *Dicer *raises the possibility that miRNAs are involved in retinal neurodegenerative disorders.

In summary, although removing *Dicer *is conceptually a crude experimental approach, the aforementioned results support the hypothesis that defects in the miRNA regulatory network in the brain are a potential cause of neurodegenerative disease.

A functional role for miRNAs in more specific neurological processes is emerging, and their dysfunction could have direct relevance for our understanding of neurodegenerative disorders [81]. This conclusion is supported by several *in vitro *examples using both gain- and loss-of-function experiments. For example, the introduction of artificial miRNAs mimicking upregulation or antisense oligonucleotides induces loss of function of primary neurons in culture [82-85].

The next challenge will be to characterize *in vivo *individual miRNAs and specific families of miRNAs in depth, which are predicted to contribute to the proper CNS function. An initial step towards this goal is the recent study of Walker and Harland. The authors show through loss-of-function experiments that in *Xenopus laevis *miR-24a is necessary for proper neural retina development by regulating apoptosis through Caspase 9 targeting [86].

## microRNAs and immune system 'diseases'

Vertebrates have evolved complex genetic programmes that simultaneously regulate the development and function of hematopoietic cells, resulting in the capacity to activate specific responses against invading foreign pathogens while maintaining self-tolerance. From recent studies, miRNAs are emerging as major players in the molecular circuitry that controls the development and differentiation of haematopoietic lineages [87].

Genetic disruption of different steps in miRNA biogenesis in mice has highlighted the key role of miRNAs during haematopoiesis. *Dicer *ablation in the T-lineage, whilst not abolishing the development of T-lymphocytes, affected their functionality [87,88]. Interestingly, ablation of *Dicer *in regulatory T cells (Treg cells) resulted in a much more severe phenotype. Mice lacking *Dicer *expression in Treg cells failed to differentiate functional Treg cells and developed a severe autoimmune disease, leading to death within the first few weeks of life [89].

Knocking-out Dicer activity in early B-cell progenitors determined a block at the pro-B cell stage during the differentiation process leading to mature activated B-cells. Gene-expression profiling revealed a miR-17-92 signature in the 3'UTRs of genes upregulated in Dicer-deficient pro-B cells; the proapoptotic molecule Bim, a top miR-17-92 target, was also highly upregulated. Surprisingly, B cell development was partially rescued by ablation of *Bim *or transgenic expression of the prosurvival protein Bcl-2 [90].

In mice the specific role of single miRNAs in the development and function of the immune system is starting to be elucidated through targeted deletion approaches. The pioneer knockout of miR-155 in mice (the first mouse knockout for a single miRNA) revealed an essential role in the acquired immunity for this miRNA. In fact, despite miR-155 null mice developed normally, immune system analysis revealed that miR-155 depletion led to pleiotropic defects in the function of B cells, T cells and dendritic cells. These mice were unable to gain acquired immunity in response to vaccination, demonstrating that miR-155 is indispensable for normal adaptive immune responses [91,92].

Another functional example derives from the study of Ventura *et al*. (2008) who demonstrated that the miR-17-92 cluster is involved in controlling B-lymphocyte proliferation. Deletion of this miRNA cluster was lethal in mice resulting in lung hypoplasia, ventricular sept defects and impairment of the pro-B to pre-B transition. Absence of miR-17-92led to increased levels of the pro-apoptotic protein Bim and inhibited B cell development at the pro-B to pre-B transition. Furthermore, while ablation of miR-106b-25 or miR-106a-363 (the two paralogous clusters) had no obvious phenotypic consequences, compound mutant embryos lacking both miR-106b-25 and miR-17-92 died at mid-gestation [93]. On the contrary, over-expression of miR-17-92 cluster in mice led to lymphoproliferative and autoimmune diseases that were associated with self-reactive antibody production [94].

Finally, Johnnidis *et al*. described the generation of a knockout mouse for miR-223, which highlighted its role in granulocyte differentiation. The myeloid-specific miR-223 negatively regulated progenitor proliferation and granulocyte differentiation. Moreover, mutant mice had an expanded granulocytic compartment resulting from a cell-autonomous increase in the number of granulocyte progenitors. These data support a model in which miR-223 acts as a fine-tuner of granulocyte production and the inflammatory response [95].

The fact that miRNAs are involved in the modulation of T cell selection, T cell receptor sensitivity as well as Treg cell development in normal immune responses, suggests that these molecules may also be involved in the development of immune system disorders of genetic origin such as immunodeficiencies or autoimmune diseases.

## microRNAs and 'diseases' affecting other tissues

In mouse, conditional inactivation of *Dicer *has been achieved in different other tissues in order to study the global function of miRNAs [23,96-101]. Using transgenes to drive Cre expression in discrete regions of the limb mesoderm, Harfe *et al*. found that removal of *Dicer *determined developmental delays, due in part to massive cell death as well as to dysregulation of specific gene expression, and brought to the formation of a much smaller limb. Strikingly, however, the authors did not detect defects in basic patterning or in tissue-specific differentiation of Dicer-deficient limb buds [23]. This class of skeletal defects was previously observed in mice with compound mutations in *Prx1 *and *Prx2 *genes [102].

To better understand the role of miRNAs in skin- and hair follicle biology, Andl and colleagues generated mice carrying an epidermal-specific *Dicer *deletion. These mice presented stunted and hypoproliferative hair follicles. Normal hair shafts were not produced in the *Dicer *mutant and the follicles lacked stem cell markers and degenerated. In contrast to decreased follicular proliferation, the epidermis became hyperproliferative. These results reveal the critical role played by Dicer in the skin and the key aspect that miRNAs may have in epidermal and hair-follicle development and function [96]. Moreover, the existence of skin-specific miRNAs involved in normal epidermal and follicular development, such as the miR-200, the miR-19 and miR-20 families, indicate that their therapeutic expression or inhibition might also be relevant to epidermal pathology [103].

To study Dicer function in the later events of lung formation, Harris and collaborators inactivated *Dicer *in the mouse lung epithelium using a *Shh*-Cre allele. As a result, the mutant lung presented a few large epithelial pouches as opposed to the numerous fine branches that are seen in a normal lung. Phenotypic differences between mutant and normal lungs were apparent, significantly, even before detection of an increase in epithelial cell death, leading the authors to propose that Dicer may play a specific role in regulating lung epithelial morphogenesis independent of its requirement in cell survival [97].

Dicer activity is essential for skeletal muscle development during embryogenesis and postnatal life. O'Rourke and colleagues (2007) showed that *Dicer *inactivation in skeletal muscle resulted in lower levels of muscle-specific miRNAs. Moreover, *Dicer *muscle mutants died perinatally and were characterized by skeletal muscle hypoplasia. Reduced skeletal muscle, in turn, was accompanied by abnormal myofibre morphology. The skeletal muscle defects associated with loss of Dicer function were explained by increased apoptosis. Furthermore, decrease in muscle mass in *Dicer *mutants was strikingly similar to the phenotypes associated with muscular dystrophies and aged skeletal muscle. This finding suggests that, in humans, *DICER *mutations, or disrupted miRNA-mediated gene regulation, should contribute to skeletal muscle myopathy and age-related sarcopenia [98].

The study of Pastorelli and co-workers [100] demonstrated that loss of *Dicer *in the developing mouse reproductive tract, under the control of *Amhr2*-Cre-mediated deletion, resulted in morphologic and functional defects in the reproductive tracts of female but not of male mice (before 3 months of age).

Recently, Sekine and colleagues have described the conditional *Dicer *ablation in the mouse liver. This resulted in prominent steatosis and in the depletion of glycogen storage. Dicer-deficient liver exhibited increased growth-promoting gene expression and robust expression of fetal stage-specific genes. The consequence of *Dicer *elimination included both increased hepatocyte proliferation and overwhelming apoptosis [101].

Finally, two different *Dicer *knockout strategies demonstrated that miRNAs are required for the development and differentiation of sensory epithelia [104] and the maintenance of the sensory neurons of the inner ear [105]. Based on studies carried out in animal models, it is clear that miRNA dysfunction may lead to severe alterations in the function of all tissues/organs that have been analysed up to now. In the majority of the aforementioned cases, the aberrant phenotypes observed are the consequence of a global impairment of miRNA processing (that is, *Dicer *knockout approaches), a condition that is highly unlikely to contribute to the pathogenesis of human genetic diseases. Nevertheless, the fact that in some organs (that is, the heart, the eye and the immune system) the dysfunction of single miRNAs may underlie phenotypes, strongly resembling those observed in human disease, suggests that miRNAs should be considered potential candidates in the pathogenesis of human genetic disorders, even monogenetic forms, likewise protein-coding genes.

## Conclusion

microRNAs are emerging as key regulators of the cell transcriptome due to their ability to finely tune gene dosage. In the last few years, they have been shown to be involved in the regulation of many cellular processes and their role in the proper differentiation and function of tissues and organs is only starting to be unravelled. It is also becoming increasingly clear that miRNAs, similarly to protein-coding genes, may harbour mutations leading to human genetic conditions, even 'classical' monogenic forms. The number of cases in which mutations in miRNAs, or in their targets, have been convincingly shown to have a pathogenic role in human genetic diseases is still limited. This may not only be explained by the recent characterization of miRNAs at the genomic level, which will now allow us to carry out the appropriate analyses, but also by the fact that the 3'UTR of mRNAs have, until recently, been generally neglected as potential sources of sequence variations with a potentially pathogenic effect in genetic diseases. However, both the improvement in experimental procedures, aimed at the identification of mutations based on efficient sequencing protocols, and the increasing knowledge in miRNA function are predicted to fill the latter gaps, underscoring the role played by miRNAs in the pathogenesis of human genetic disorders in the coming years.

## Abbreviations

CNS: central nervous system; DCM: dilated cardiomyopathy; miRNA: microRNA; mRNA: messenger RNA; nt: nucleotides; pre-miRNA: precursor miRNS; RISC: RNA-induced silencing complex; RNP: ribonucleoprotein; SNP: single nucleotide polymorphism; ss: single-stranded; Tg: transgenic; UTR: untranslated region; VSD: ventricular sept defects.

## Competing interests

The authors declare that they have no competing interests.

## Authors' contributions

NM, VAG and SB wrote the review manuscript. The authors read and approved the final manuscript.
